# Involvement of cancer-derived EMT cells in the accumulation of ^18^F-fluorodeoxyglucose in the hypoxic cancer microenvironment

**DOI:** 10.1038/s41598-021-88414-1

**Published:** 2021-05-17

**Authors:** Sachi Sugita, Masanori Yamato, Toshimitsu Hatabu, Yosky Kataoka

**Affiliations:** 1 Laboratory of Animal Physiology, Graduate School of Environmental and Life Science, Okayama University, Okayama, Okayama 700-8530 Japan; 2 Laboratory for Cellular Function Imaging, RIKEN Center for Biosystems Dynamics Research, 6-7-3 Minatojima-minamimachi, Chuo-ku, Kobe, Hyogo 650-0047 Japan; 3 Multi-Modal Microstructure Analysis Unit, RIKEN-JEOL Collaboration Center, 6-7-3 Minatojima-minamimachi, Chuo-ku, Kobe, Hyogo 650-0047 Japan

**Keywords:** Cancer, Oncology

## Abstract

A high rate of glycolysis, one of the most common features of cancer, is used in positron emission tomography (PET) imaging to visualize tumor tissues using ^18^F-fluorodeoxyglucose (^18^F-FDG). Heterogeneous intratumoral distribution of ^18^F-FDG in tissues has been established in some types of cancer, and the maximum standardized uptake value (SUVmax) has been correlated with poor prognosis. However, the phenotype of cells that show high ^18^F-FDG accumulation in tumors remains unknown. Here, we combined quantitative micro-autoradiography with fluorescence immunohistochemistry to simultaneously visualize ^18^F-FDG distribution, the expression of multiple proteins, and hypoxic regions in the cancer microenvironment of a human A431 xenograft tumor in C.B-17/Icr-*scid*/*scid* mice. We found that the highest ^18^F-FDG accumulation was in cancer-derived cells undergoing epithelial-mesenchymal transition (EMT) in hypoxic regions, implicating these regions as a major contributor to increased glucose metabolism, as measured by ^18^F-FDG-PET.

## Introduction

The most commonly used positron emission tomography (PET) imaging agent for staging purposes, and for the monitoring of malignant tumors is ^18^F-fluorodeoxyglucose (^18^F-FDG). It is a glucose analog transported into the cells by glucose-transporters (GLUT) via a passive process similar to that involved in glucose transport. ^18^F-FDG transport is dependent on the cytosolic glucose concentration (glucose demand)^1^. Once transported into tumor cells, ^18^F-FDG is retained as ^18^F-FDG-6-phosphate after phosphorylation by hexokinase^2^. PET imaging with ^18^F-FDG is used to monitor the glucose demand in tissues mainly for cancer diagnosis. The uptake of ^18^F-FDG by a tissue is quantified in terms of the standardized uptake value (SUV), which is the ^18^F-FDG uptake rate corrected for the body mass and the radioactivity of the injected substance^3^.

Over the past several years, accumulating clinical data from ^18^F-FDG-PET imaging in patients with cancer has shown that SUV_max_, the highest SUV of ^18^F-FDG in the region of interest (ROI), correlates well with the malignancy of cancer^4–8^. For example, the progression-free survival rate and overall survival rate were inversely correlated with SUV_max_ in limited-stage small cell lung cancer, renal cell carcinoma, and squamous cell carcinoma of the head and neck^9–11^. The risks of metastasis and recurrence in oral squamous cell carcinoma have also been correlated to SUV_max_^7^. These findings suggest that the high accumulation of ^18^F-FDG in cells can be linked to the aggressiveness of cancer.

Recently, the cancer microenvironment has been found to be associated with tumor glycolysis. Heiden et al. reported that the expression of prediction factors, including those that indicate hypoxia and the epithelial-mesenchymal transition (EMT), is correlated with SUV_max_ in cases of operable esophageal adenocarcinoma^12^. Furthermore, Iyer et al. and Seagroves et al. have previously reported that the hypoxia-inducible factor-1 (HIF-1), a heterodimeric transcription factor, regulates the activation of genes encoding glucose transporters and glycolytic enzymes^13,14^. Pavlides et al. proposed a two-compartment model called the “reverse Warburg effect,” which explains how cancer cells use metabolism to interact with cancer-associated fibroblasts (CAFs)^15–17^. In this model, reactive oxygen species (ROS) produced by cancer cells give rise to CAFs by acquiring metabolic features that are different from those of normal fibroblasts. CAFs produce high-energy fuels, such as lactate, ketone bodies, and fatty acids, by aerobic glycolysis, through the upregulation of HIF-1. Cancer cells can utilize these high-energy fuels from CAFs by oxidative phosphorylation^17^. Furthermore, S100A4, a member of the S100 calcium-binding protein family, is a CAF-related protein^18^. In many types of cancers, S100A4 expression is correlated with a poor prognosis^19^. This suggests that CAFs contribute to high ^18^F-FDG accumulation and are involved in the progression of cancer. On the contrary, it has also been reported that EMT is amplified by the overexpression of S100A4 in cancer cells^20–23^. Shangguan et al. reported that a high SUV_max_ of ^18^F-FDG is associated with the hypoxic microenvironment of the tumor site and intensive S100A4 (also defined as fibroblast-specific protein 1- FSP1) expression^24^.

These findings led us to the hypothesis that the high accumulation of ^18^F-FDG is induced by CAFs or cells undergoing EMT in the hypoxic region of the cancer microenvironment. Identifying characteristics of cells that show the highest ^18^F-FDG uptake may contribute to the identification of drug targets that facilitates cancer malignancy. Despite several ^18^F-FDG imaging studies on tumors consisting of heterogeneous cell types, the role of metabolic heterogeneity among various cell types in the accumulation of ^18^F-FDG has not been addressed so far, mainly due to the low spatial resolutions of the existing methods^25–27^. Previously, we have visualized ^18^F-FDG uptake and inflammatory protein expression simultaneously at the cellular level of spatial resolution by combining micro-autoradiography and immunohistochemistry^28^. In this study, we employed a quantitative micro-autoradiographic technique along with fluorescence histochemistry in mice with squamous cell carcinoma to examine the correlation between ^18^F-FDG -SUV_max_ and malignancy by simultaneously visualizing ^18^F-FDG distribution, expression of multiple proteins, and the hypoxic regions in the cancer micro-environment.

## Results

**Figure 1:**
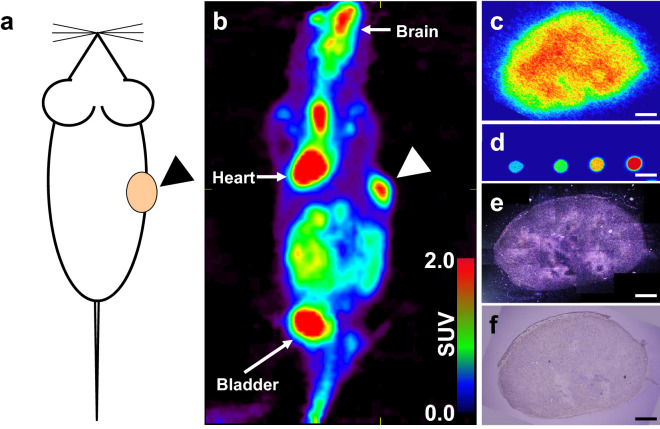
Multi-modal imaging of ^18^F-FDG distribution in the A431 cell-derived tumor. (**a**) Illustration of tumor xenograft mouse. The arrow-head shows the location of the A431 cell-derived tumor. (**b**) Imaging to visualize tumor tissues using ^18^F-fluorodeoxyglucose positron emission tomography (^18^F-FDG PET) coronal view image of the A431 xenograft SCID mouse. The arrow-head shows the A431 cell-derived tumor. (**c**) Macro-autoradiography of an A431 cell-derived tumor section. (**d**) Macro-autoradiography of diluted dosage. The spots show 1 μL of 1:200-, 1:400-, 1:800-, and 1:1600-diluted injected dosages, from right to left. (**e**) The tiling image of micro-autoradiographs of a whole section adjacent to (**c**) in dark field observation. (**f**) HE staining of an adjacent section of (**c**). Bars: 1000 μm.

As shown in Fig. 1, PET imaging revealed the intratumoral distribution of ^18^F-FDG in a xenograft mouse that was inoculated with A431 cells (Fig. 1a,b). Intensive radioactivity was observed in the xenograft tumor as well as in the brain, heart, and bladder. Macro-autoradiographic images (Fig. 1c) of the tumor showed a heterogeneous distribution of radioactivity in the tissue, suggesting heterogeneous intratumoral distribution of ^18^F-FDG due to the distinct metabolic features of the cells in the tumor. Radioactivity was estimated based on macro-autoradiographic images of 1:200-, 1:400-, 1:800-, and 1:1600-diluted ^18^F-FDG solutions (Fig. 1d). Micro-autoradiography was used to visualize the distribution of silver grains formed by ^18^F-FDG (Fig. 1e,f). Micro-autoradiography, macro-autoradiography, and hematoxylin & eosin (HE) staining were performed using adjacent slices of the same tumor. The grain density observed in micro-autoradiography was highly consistent with the radioactivity data obtained through macro-autoradiography.

**Figure 2:**
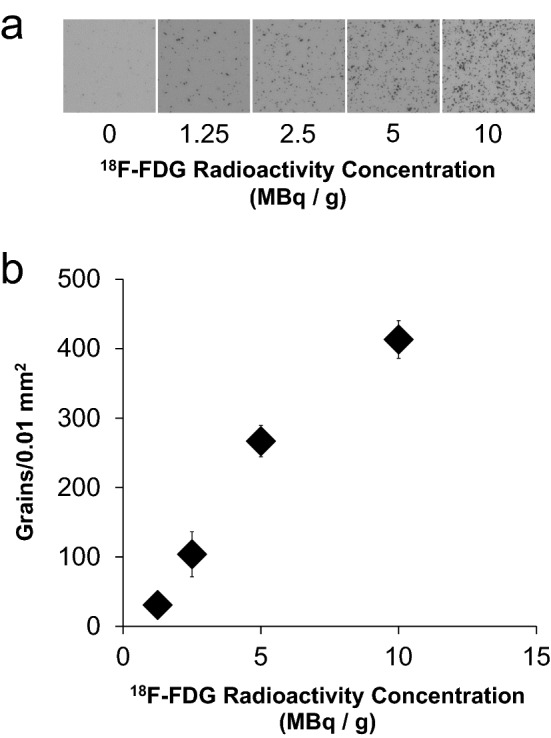
The quantitative radioactive range of micro-autoradiography. (**a**) Micro-autoradiographs of various concentrations of ^18^F-FDG-containing samples sliced to 10 μm thickness. (**b**) Scatter plot of grain counts visualized at each concentration of micro-autoradiography. (n = 4, mean ± S.D.).

Figure 2 shows the quantitative capability of micro-autoradiography. The grain density increased in correspondence with the radioactivity concentration (Fig. 2a). Grain counts between 1.25 MBq/g and 5 MBq/g showed a good correlation with the radioactivity. At 10 MBq/g, the correlation became weaker (Fig. 2b), probably due to the merging of the silver grains. The grain counts were less than 5 MBq/g in the micro-autoradiography experiments, indicating that the data were quantitatively reliable.

**Figure 3:**
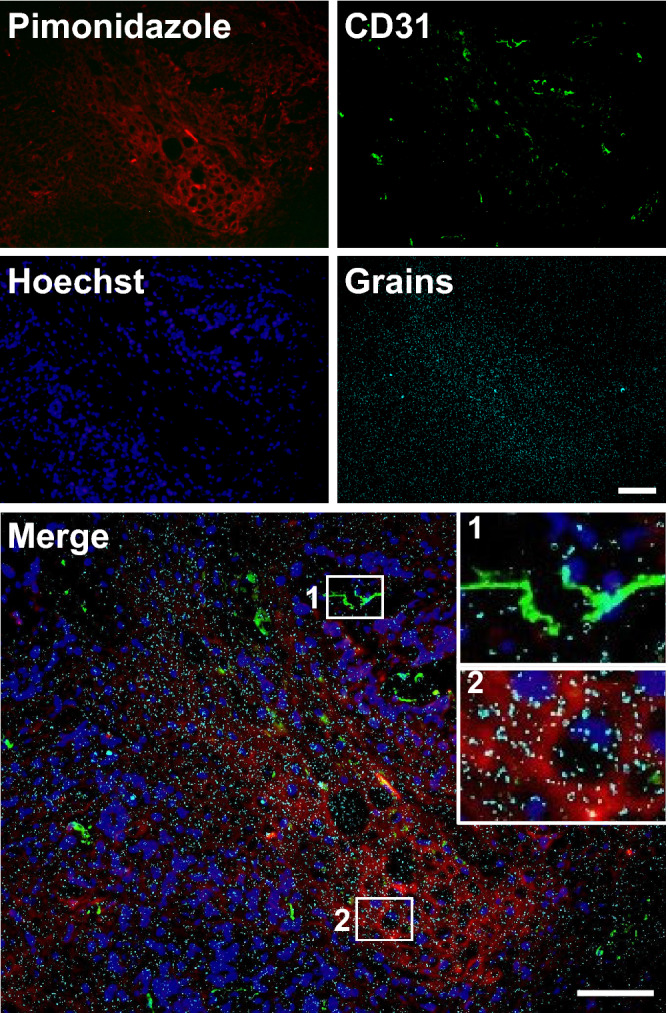
Micro-autoradiography of ^18^F-FDG combined with immunohistochemistry. Merged image shows pimonidazole (red), CD31 (green), nuclei (blue), and grains (cyan) in the same view of the A431 cell-derived tumor section. Insets show a magnified view of the region demarcated by a white square in each panel. Bars: 100 μm.

Additionally, we combined micro-autoradiography to visualize ^18^F-FDG uptake with immunohistochemistry for pimonidazole and CD31 to visualize hypoxic regions and infiltrated blood vessels, respectively, in the same tumor section (Fig. 3). The immunohistochemical analysis results indicated that pimonidazole-positive regions were observed in avascular areas of the tumor. Of note, although CD31-positive cells were found in pimonidazole-positive areas, those cells did not compose the vasculature. The lower nuclear density in the pimonidazole-positive area indicated a lower cell division activity in the hypoxic area than in the normoxic area. Moreover, the significantly high grain density in the pimonidazole-positive area suggested that ^18^F-FDG uptake was high in the hypoxic areas.

**Figure 4:**
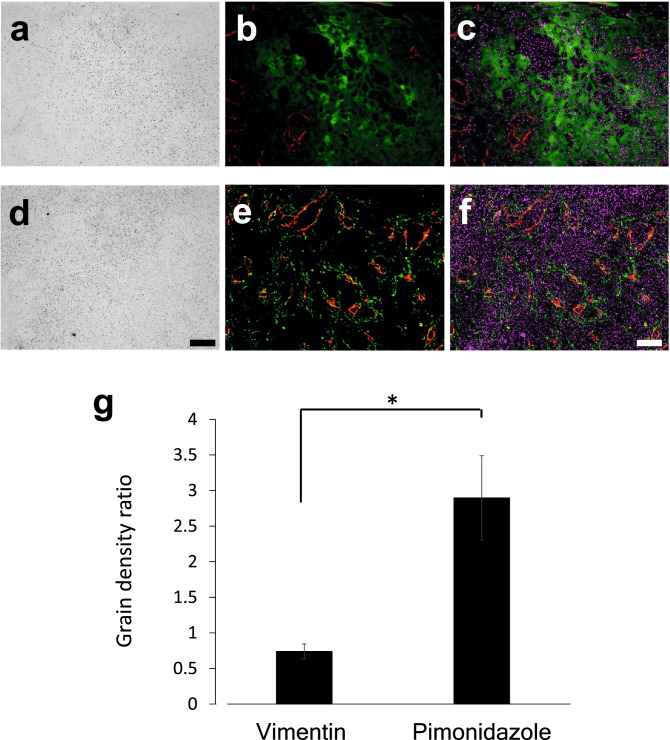
^18^F-FDG accumulation in hypoxic cells and host-derived cells. (**a**) Micro-autoradiography, (**b**) Merged image for pimonidazole (green) and CD31 (red), and (**c**) merged image for pimonidazole (green), CD31 (red), and grains (magenta) in the same view of the A431 cell-derived tumor section. (**d**) Micro-autoradiography, (**e**) Merged image for vimentin (green) and CD31 (red), and (**f**) Merged image for vimentin (green), CD31 (red), and grains (magenta) in the same view. (**g**) Grain density ratio of the pimonidazole-positive region to the CD31-positive region and the vimentin-positive region to the CD31-positive region (n = 3, mean ± SEM, Student’s *t*-test, two-sided, *: *p* < 0.05). Bars: 100 μm.

Hypoxia-mediated induction of host cell infiltration has been widely reported^29,30^. To determine the ^18^F-FDG uptake by infiltrated host mesenchymal cells, blood vessels, and hypoxic cells, we employed micro-autoradiography in combination with immunohistochemistry for vimentin, CD31, and pimonidazole (Fig. 4). The pimonidazole-positive area showed low CD31-positive vasculature, with high grain density (Fig. 4a–c). Many vimentin-positive cells were found near the CD31-positive area, indicating lumen formation (Fig. 4e). However, the distribution pattern of vimentin-positive cells was not correlated with grain density (Fig. 4d–f). To compare the grain density of the pimonidazole- and vimentin-positive regions detected in separate slices, the density of the CD31-positive region was used as an internal control. The grain density ratio of the pimonidazole-positive region to the CD31-positive region was significantly higher than that of the vimentin-positive region to the CD31-positive region (Fig. 4g). As the anti-vimentin antibody used could react with both human and mouse vimentin, the expression of mouse S100A4 in vimentin-positive cells was confirmed. Moreover, the vimentin-positive cells coincided with the mouse-S100A4 positive cells (Supplementary Fig. 1). These results indicated that the hypoxic cancer microenvironment contributed to ^18^F-FDG accumulation, whereas host-derived vimentin- and CD31-positive cells did not.

**Figure 5:**
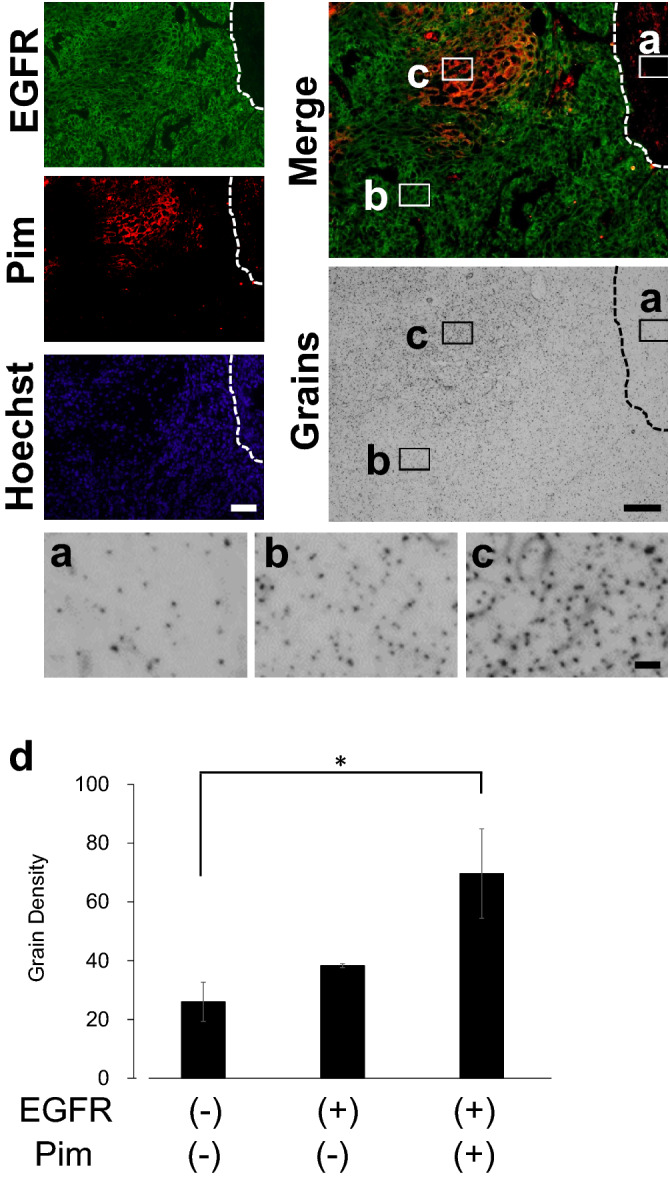
^18^F-FDG accumulation in hypoxic cells expressing EGFR. The dotted line shows the boundary between the tumor and the skin area. (**a**) Magnified view of the micro-autoradiography image showing the pimonidazole- and EGFR-negative skin area, (**b**) pimonidazole-negative and EGFR-positive area, and (**c**) pimonidazole- and EGFR-positive area. (**d**) Determination of grain density in the pimonidazole- and EGFR-positive and negative areas, focusing on the same-sized ROIs (n = 3, mean ± SEM, Tukey’s multiple comparison, *: *p* < 0.05). Pim: Pimonidazole. Bars: 100 μm (EGFR, Pimonidazole, Hoechst, Merge and Silver grains), 10 μm (**a**–**c**).

A431 tumors were reported to express EGFR and HER2^31^. Next, we examined the distribution profile of ^18^F-FDG-accumulating hypoxic cells by combining micro-autoradiography with immunohistochemistry for EGFR, an A431 cell-derived tumor cell marker, and pimonidazole in the same tumor slices (Fig. 5). The tissue slices contained areas with EGFR-immunopositive cancer cells and pimonidazole-positive cells. We quantified the ^18^F-FDG uptake in each area by counting the silver grains in each ROI (Fig. 5a–c). The grain-count was higher in the EGFR-positive hypoxic area than in the EGFR-negative non-tumor tissue area. Tukey test showed a significant difference between these two areas (*p* = 0.0416). Within the EGFR-positive area, the pimonidazole-positive hypoxic area showed a 1.8-fold higher grain-count than the pimonidazole-negative normoxic area. These results were consistent with those obtained using the “computer-aided fluorescence positive area” method (Supplementary Fig. 2). Liu et al. reported that hypoxia decreases EGFR expression in MCF-7 and HeLa xenograft tumors^32^. However, in the A431 xenograft tumor, we found that EGFR expression was decreased in the pimonidazole-positive area (Fig. 5), whereas HER2 expression was increased in EGFR decreased area (Supplementary Fig. 3). The number of silver grains in the HER2-positive area was 1.8-fold higher than that in the EGFR-positive area (Supplementary Fig. 6). These results demonstrated that ^18^F-FDG was markedly accumulated in hypoxic A431 tumor cells tending to express HER2.

**Figure 6:**
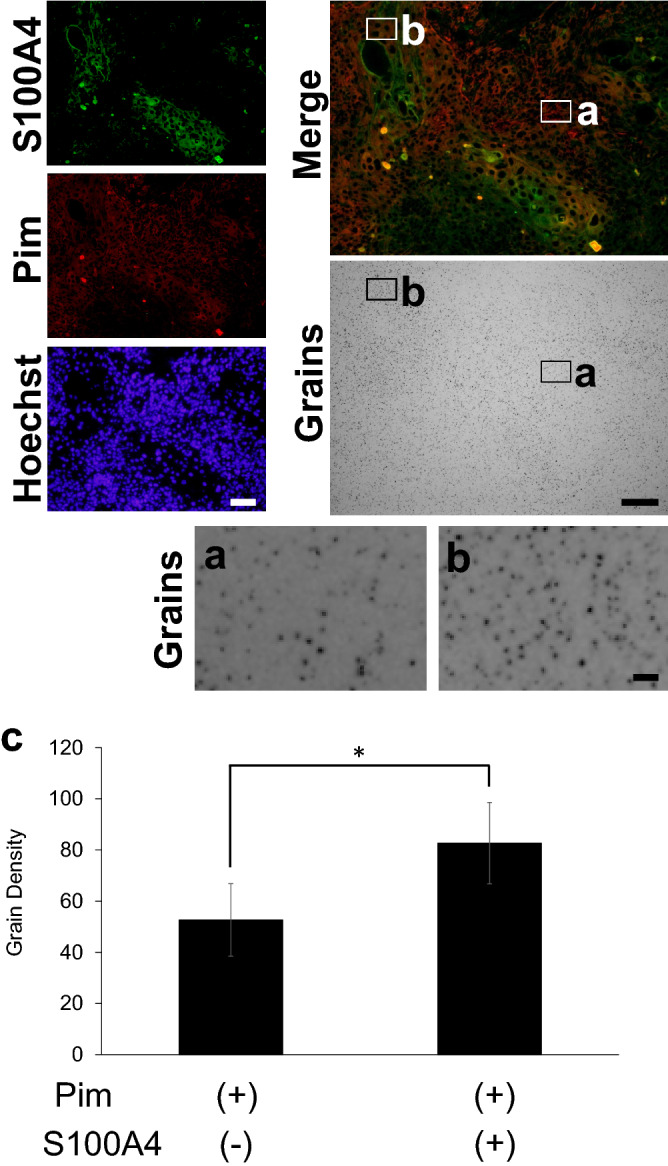
^18^F-FDG accumulation in hypoxic tumor cells expressing human S100A4. (**a**) Magnified view of micro-autoradiographic image showing the pimonidazole-positive and S100A4-negative area, and (**b**) the pimonidazole-positive and S100A4-positive area. (**c**) Determination of grain density in pimonidazole-positive and S100A4- negative or positive area focusing on ROIs of the same size (n = 3, mean ± SEM, Student *t*-test, one-sided, *: *p* < 0.05). Pim: Pimonidazole. Bars: 100 μm (S100A4, Pimonidazole, Hoechst, Merge and Silver grains), 10 μm (**a**,**b**).

Subsequently, we investigated the relationship between pimonidazole-positive hypoxic cells, human S100A4-positive cells, and ^18^F-FDG accumulation (Fig. 6). Within the pimonidazole-positive hypoxic tumor cell area, the grain-count was 1.6-fold higher in the human S100A4-positive area than in the human S100A4-negative area (Fig. 6c). Moreover, a statistically significant difference in human S100A4 expression (*p* = 0.0483) was observed between these regions. These results were consistent with those obtained using the “computer-aided fluorescence positive area” counting method (Supplementary Fig. 5). The results showed that cancer-derived EMT cells had the highest ^18^F-FDG uptake in the A431 xenograft tumor.

## Discussion

To the best of our knowledge, this is the first report describing the simultaneous quantitative visualization of ^18^F-FDG distribution and the tumor microenvironment by combining fluorescence immunohistochemistry and micro- and macro-autoradiography. Increased glucose uptake and a high rate of glycolysis are the most common metabolic changes in cancer cells^33^. Several studies have identified and assessed canonical and non-canonical glycolytic pathways to develop novel cancer treatment strategies targeting cancer-specific metabolisms^34–36^. However, most glycolysis inhibitors have not succeeded as therapeutic modalities because of their systemic toxicity^35^. The success of cancer therapy using glycolysis inhibitors has been elusive due to our limited knowledge about high glucose demanding cells. We have shown that cancer-derived hypoxic S100A4-positive cells accumulate higher levels of ^18^F-FDG than other normoxic and hypoxic cancer cells from the A431 cell-derived tumor. The results of our study indicate that cells undergoing EMT are mainly involved in glucose metabolism in cancer.

Huang et al. demonstrated a correlation between the uptake of ^18^F-FDG and the accumulation of pimonidazole in a tumor xenograft mouse model, using ^18^F-FDG macro-autoradiography^26^. Using in vitro and in vivo studies, Shen et al. reported that intratumoral ^18^F-FDG distribution is microenvironment-dependent because hypoxic cancer cells (pimonidazole-positive) need more ^18^F-FDG than normoxic cancer cells^37^. In the present study, we performed a quantitative analysis of ^18^F-FDG accumulation in cells that were positive or negative for EGFR and/or pimonidazole. Hypoxic regions were observed in the EGFR-positive areas. Our results demonstrate that, at the cellular level, hypoxia is responsible for ^18^F-FDG accumulation in cancer, rather than normoxia. As the transport of ^18^F-FDG across the cell membrane is mediated by a transporter, its expression pattern influences the accumulation of ^18^F-FDG. GLUT1 is one of the members of the SLC2 membrane transporter family which predominantly transport substrates such as glucose, galactose, mannose, and glucosamine. Higashi et al. explored the correlation between the expression of GLUT1 and the SUV_mean_ of ^18^F-FDG in patients diagnosed with non-small cell lung cancer^38^. Of note, GLUT1 has been used to estimate the hypoxic environment as the *SLC2A1* gene (coding for GLUT1) is activated by HIF1α^39^. In the present study, however, we used pimonidazole as the hypoxia marker. We used this approach because GLUT1 distribution was not completely consistent with the HIF1α-positive area or pimonidazole-positive area in the context of ^18^F-FDG uptake in A431 tumors (Supplementary Fig. 7). Of note, GLUT1 was intensively expressed not only in the hypoxic area but also in CD31-positive vasculature (angiogenesis in the tumor tissues).

Furthermore, we analyzed the correlation between ^18^F-FDG accumulation and infiltrated host-derived cells and cancer-derived cells. The distribution shown by CD31 and pimonidazole around the vasculature was consistent with the result reported by Lee et al.^40^. Although this observation regarding grain distribution was consistent with the results of macro-autoradiography reported by Shangguan et al.^24^, this is the first study to demonstrate high ^18^F-FDG uptake using micro-autoradiography in combination with analysis of pimonidazole distribution. Our data shows that HER2 amplification was observed in regions showing a loss of EGFR expression; anti-EGFR therapy has been therefore used as a basis for targeting tumors expressing EGFR ^41–44^. These results may explain the correlation between high SUV_max_ in ^18^FDG-PET and resistance to EGFR inhibitor or EGFR-TK inhibitor in clinical studies. The ^18^F-labeled nitroimidazole reagent ^18^F-FMISO is widely used for imaging hypoxic regions in cancer. Studies have shown that ^18^F-FDG SUV_max_ can be correlated with ^18^F-FMISO TBR_max_ in head and neck cancer^45,46^. However, it is widely known that the distribution pattern of ^18^F-FDG is not completely consistent with that for ^18^F-FMISO, despite the correlation between ^18^F-FDG distribution and hypoxia marker expression. In this study, we have shown that ^18^F-FDG biodistribution in the hypoxic tumor area was not uniform.

In this study, hypoxic human-S100A4 positive cells showed higher ^18^F-FDG accumulation than in vimentin and CD31 positive cells. Misra et al. have demonstrated that hypoxia induces a decrease in the epithelial cell marker E-cadherin and the loss of cell–cell interactions in A431 cells in vitro^47^. These results indicate that the major ^18^F-FDG-accumulating cells in the A431 cell-derived tumor were hypoxic human S100A4-positive cells derived from the inoculated cancer cells and not vimentin-positive CAFs, including resident fibroblasts, bone marrow cells, adipocytes, endothelial cells, and epithelial cells. In the reverse Warburg Effect model, ROS from normoxic cancer cells cause metabolic changes, including the upregulation of glycolysis-related kinases, through the elevation of HIF-1α in the surrounding CAFs^48^; this indicates that hypoxia can also affect glycolysis. We believe that the increment of glucose demand in tumor cells with EMT, which is accompanied by human S100A4 expression under hypoxia, can be measured as SUV_max_ in A431 inoculated mouse ^18^F-FDG-PET. In this study, we used a xenograft model to define cancer-derived and host-derived cells clearly, although the glycolysis occurring in immune cells infiltrated into a tumor was not evaluated.

Our data in the present study suggest that hypoxic cancer cells undergoing EMT are the main contributors to SUV_max_, which is highly correlated with malignancy in the clinical context^4–8^. Thus, hypoxic cancer cells undergoing EMT may be potential targets for cancer therapy. Until now, many types of anti-cancer drugs have been developed. However, the intra-tumoral distribution of anti-cancer drugs has been often reported to be limited around the vasculature; their distribution into the hypoxic area is limited^49^. The reason for such a restricted distribution may be relevant to the fluid pressure elevation caused by abnormal angiogenesis^50^. Hence, a novel strategy to deliver anti-cancer drugs into hypoxic regions, such as regions with increased fluid pressure, will aid in cancer therapy.

In conclusion, our study adds new and important insights into the role of EMT cells in causing high ^18^F-FDG accumulation in cancer and provides a new research tool for target discovery in cancers.

## Methods

### Cell culture

The human A431 cell line was purchased from American Type Culture Collection. Cells were cultured in high glucose (4.5 g/L) Dulbecco's Modified Eagle Medium (DMEM) (Nacalai Tesque) supplemented with 10% fetal bovine serum (Thermo Scientific) and a mixture of 100 units/mL penicillin and 100 mg/mL streptomycin (Gibco). Cells were grown in a humidified incubator, in a 37 °C air atmosphere, containing 5% CO_2_ and 95% air. Exponentially growing cells were harvested and treated with 0.25% (w/v) trypsin and 1 mM ethylenediaminetetraacetic acid solution (Nacalai Tesque), and were suspended in phosphate-buffered saline (PBS).

### In vivo xenograft tumor experiments

All experiments were performed using 4 to 6-week-old female C.B-17/Icr-*scid*/*scid* jcl mice purchased from CLEA Japan, Inc. All experimental protocols were approved by the Ethics Committee on Animal Care and Use, RIKEN Center for Biosystems Dynamics Research (MAH21-19-8) and were performed as per the Principles of Laboratory Animal Care. Animal care and use are confirmed in compliance with the ARRIVE guidelines. The mice were housed (4–5 animals in each cage) at a constant temperature (22–23 °C), humidity (50%-60%), and were maintained under a 12 h light–dark cycle (lights off at 8:00 PM), with access to food and water ad libitum. The mice were subcutaneously injected with 1 × 10^6^ A431 cells in 0.1 mL of PBS into the flank while awake. All experiments were performed on the day the major axis of the tumor reached 5–10 mm in size (14 days after injection).

### ^18^F-FDG preparation

^18^F-FDG was provided by the Division of Molecular Imaging at the Institute of Biomedical Research and Innovation. Briefly, ^18^F was produced by the nuclear reaction of ^18^O(p,n)^18^F using a cyclotron system (Cypris HM-18; Sumitomo Heavy Industries, Ltd. Tokyo, Japan). Following the method reported by Hamacher et al.^51^, with a minor modification, ^18^F-FDG was automatically synthesized, within 40 min, using a radiochemical system (F-200; Sumitomo Heavy Industries, Ltd. Tokyo, Japan).

### Small animal ^18^F-FDG PET imaging

Small-animal PET imaging studies were performed using MicroPET Focus 220 scanners (Siemens Healthineers, Tokyo, Japan). The A431 xenografted mice were anesthetized with a mixture of 1.5% isoflurane and N_2_O:O_2_ (7:3) gas and were placed on a heating pad to maintain their body temperature at 37 °C during the PET scan. The mice were intravenously injected with ^18^F-FDG (7.4 MBq) through the tail vein, 15 min after they were anaesthetized^28^. Thirty min after ^18^F-FDG injection, the emission data were acquired for 30 min, using a two-dimensional list-mode method. The data were reconstructed with a filtered back-projection algorithm, using the ASIPro VM (version 6.0; Concorde Microsystems) and PMOD (version 3.4; PMOD Technologies LLC) software. Regional uptake of radioactivity in organs was decay-corrected based on injection time and expressed as the standardized uptake value (SUV), where SUV = tissue radioactivity concentration (MBq/cm^3^)/radioactivity of the injected substance (MBq) × body weight (g). The PET images are shown in rainbow false color.

### Macro-autoradiography and micro-autoradiography

Macro-autoradiography and micro-autoradiography were performed using a modified method described in our previous study, as reported by Yamato et al.^28^. Briefly, A431 xenograft mice were intravenously injected with ^18^F-FDG (300 MBq/200 μL). Animals were euthanized 45 min after the ^18^F-FDG administration under deep anesthesia induced by pentobarbital. All blood was withdrawn by perfusing saline through the left ventricle. The tumor was quickly dissected and frozen on dry ice. For macro-autoradiography, tissue sections of 5 μm thickness were prepared and mounted on glass slides; 1 μL of 1:200, 1:400, 1:800, and 1:1600-diluted injected dose spotted paper and tissue sections were contacted with an imaging plate (BAS IP SR 2040E; GE Healthcare Japan) for 60 min. The imaging plate was scanned with a bioimaging analyzer (FLA-7000; GE Healthcare Japan), and the macro-autoradiograph images were displayed using the Image Gauge software (version 4.21; Fuji Photo Film). For micro-autoradiography, tissue sections of 5 μm thickness were prepared and mounted on glass slides coated with NTB2 nuclear emulsion (Kodak) that was diluted 13:7 with distilled water. The coated slides were immediately frozen on a dry ice block and kept in exposure boxes cooled with dry ice. After a 6 h exposure, the sections were developed in a D-19 developer (Kodak) for 5 min, fixed in Fuji Fix (Fuji Photo Film) for 15 min at room temperature, and then washed in water for 10 min. The micro-autoradiographs were photographed using a microscope (IX-71; OLYMPUS) equipped with a digital camera (DP-71; OLYMPUS) and DP controller software (version 3.1.1.267; OLYMPUS) under a bright or dark field observation. The radioactivity of the tumor slices was quantified using a gamma-counter (1480 WIZARD; PerkinElmer).

### Standard curve of micro-autoradiography

^18^F-FDG (20 MBq/50 μL) was mixed with O.C.T compound (1.0 g; Sakura; 45,833) and frozen with dry ice. Sections (5 μm thick) were made and mounted at about 110 min, 220 min, 330 min, and 440 min after mixing on coated slides. Four h after mounting, the sections were exposed, developed, and subjected to bright-field photomicrography, as described earlier in the macro-autoradiography and micro-autoradiography section. The grain particles per 0.01 mm^2^ of the photomicrographs were binarized with a variable threshold so that the background silver grains were not recognized. With the binarized image, the silver grains were counted. The ImageJ software (version 1.48v; National Institutes of Health, Bethesda, Maryland, U.S.A.) was used for the photomicrograph analysis.

### Micro-autoradiography combined with immunohistochemistry of tumor sections

In all, nine mice were used for micro-autoradiography combined with immunohistochemistry. A431 xenografted mice were injected intravenously with a mixture of ^18^F-FDG (300 MBq) and pimonidazole-HCl (1200 μg; Hypoxyprobe; Hypoxyprobe-1) in 0.1 mL of physiological saline. Micro-autoradiography was performed as described in the macro-autoradiography and micro-autoradiography section. After the sections were incubated with blocking solution (5% normal serum in PBS-T) at room temperature for 1 h, they were incubated with antibodies against vimentin (mesenchymal cell marker, 1:200; Synaptic Systems; cat. no. 172 002), pimonidazole (1:100; Hypoxyprobe; cat. no. PAb2627AP), CD31 (endothelial cell marker, 1:50; Santa Cruz Biotechnology; cat. no. sc-101454), human EGFR (A431 derived tumor cell marker, 1:200; R&D systems; cat. no. AF231), human S100A4 (1:50; Acris Antibodies; cat. no. AP16925PU-N), and mouse S100A4 (1:50; R&D systems; cat. no. MAB4138) at 4 °C, overnight. After the sections were washed in PBS-T three times, they were incubated with Cy2, Cy3, or Cy5 labeled anti-rat, anti-goat, or anti-rabbit IgG (Vector Laboratories; 1:200 dilution) at room temperature for 3 h. The sections were washed with PBS-T thrice again. The nuclei were visualized using Hoechst 33342 (AAT Bioquest; 17530). The sections were photographed using a microscope (IX-71; OLYMPUS) equipped with a digital camera (DP-71; OLYMPUS) and DP controller software (version 3.1.1.267; OLYMPUS) in the bright field or fluorescence observation mode. The quantitative analysis of micro-autoradiographs was conducted using the ImageJ software (version 1.48v).

Grain counts in the pimonidazole-, EGFR-, HER2-, and human S100A4-positive areas were obtained from 4073 μm^2^ square regions of interest (ROIs), which were drawn on the fluorescence positive or negative area in fluorescence photomicrographs. Silver grains were counted visually or using a computer-aided method. The computer-aided grain counting was conducted by binarizing the bright-field photomicrographs with an appropriate threshold and counting the particles with an area less than 40 pixels. The ROI size was determined by at least fitting the smallest pimonidazole-positive area. The ROIs included the nuclei and cytosols of fluorescence positive cells. The photomicrograph showed the immunofluorescence-positive or negative areas of regions randomly taken from the cancer of an individual mouse. To verify the counting methods used, grain counting with computer-aided ROIs was also conducted for pimonidazole-, EGFR-, HER2-, and S100A4-positive areas. In these analyses, the unlabeled nucleus and cytosol of fluorescence-positive cells were filled using a 20 × 20 pixels mean-filter. As the vimentin and CD31-positive areas were too small to analyze, the grain counts with 4073 μm^2^ ROIs and computer-aided ROIs were used. In these cases, grain counts for the vimentin-, CD31- and pimonidazole-positive cells were obtained by overlaying binary fluorescence photomicrographs, which showed a fluorescence positive area on the bright field photomicrograph. All binary images were produced using ImageJ software, and the binary thresholds were validated visually.

### Statistical analysis

Statistical analysis was performed using Excel (Microsoft Office 2013) or JMP (SAS Institute Inc., ver. 14.2.0). Parametric data are presented as mean ± SEM. Significance was determined using the Tukey’s multiple comparison and Student’s *t*-tests. A *P*-value of less than 0.05 was considered to be statistically significant.

## Supplementary Information

Supplementary Information

## Data Availability

The datasets generated during and/or analysed during the current study are available from the corresponding author on reasonable request.

## References

[1] Mueckler M, Thorens B (2013). The SLC2 (GLUT) family of membrane transporters. Mol. Aspects Med..

[2] Maschauer S, Prante O, Hoffmann M, Deichen JT, Kuwert T (2004). Characterization of 18F-FDG uptake in human endothelial cells in vitro. J. Nucl. Med..

[3] Zasadny KR, Wahl RL (1993). Standardized uptake values of normal tissues at PET with 2-[fluorine-18]-fluoro-2-deoxy-D-glucose: variations with body weight and a method for correction. Radiology.

[4] Lodge MA (2017). Repeatability of SUV in Oncologic (18)F-FDG PET. J. Nucl. Med..

[5] de Langen AJ (2012). Repeatability of 18F-FDG uptake measurements in tumors: a metaanalysis. J. Nucl. Med..

[6] Murata H (2018). SUV(max)-based parameters of FDG-PET/CT reliably predict pathologic complete response after preoperative hyperthermo-chemoradiotherapy in rectal cancer. Anticancer Res..

[7] Morand GB (2018). Maximum standardized uptake value (SUV(max)) of Primary tumor predicts occult neck metastasis in oral cancer. Sci. Rep..

[8] Huang YC (2017). FDG PET using SUV(max) for preoperative T-staging of esophageal squamous cell carcinoma with and without neoadjuvant chemoradiotherapy. BMC Med. Imaging.

[9] Kwon SH (2016). The highest metabolic activity on FDG PET is associated with overall survival in limited-stage small-cell lung cancer. Medicine (Baltimore).

[10] Ito H (2017). One-month assessment of renal cell carcinoma treated by everolimus using FDG PET/CT predicts progression-free and overall survival. Cancer Chemother. Pharmacol..

[11] Šedienė, S., Kulakienė, I., Rudžianskas, V. & Ambrazienė, R. The Role of 18-Fluoro-2-Deoxy-Glucose Positron Emission Tomography/Computed Tomography as Response and Prognosis Predictive Factor of Concurrent Chemoradiotherapy after Induction Chemotherapy in Head and Neck Squamous Cell Carcinoma: A Prospective Study. *Medicina (Kaunas)* **54**, 31 (2018). 10.3390/medicina54020031 PMC6037264 30344262

[12] Heiden BT (2018). Positron emission tomography 18F-fluorodeoxyglucose uptake correlates with KRAS and EMT gene signatures in operable esophageal adenocarcinoma. J. Surg. Res..

[13] Iyer NV (1998). Cellular and developmental control of O_2_ homeostasis by hypoxia-inducible factor 1 alpha. Genes Dev..

[14] Seagroves TN (2001). Transcription factor HIF-1 is a necessary mediator of the pasteur effect in mammalian cells. Mol. Cell Biol..

[15] Kalluri R (2016). The biology and function of fibroblasts in cancer. Nat. Rev. Cancer.

[16] Chen X, Song E (2019). Turning foes to friends: targeting cancer-associated fibroblasts. Nat. Rev. Drug Discov..

[17] Pavlides S (2009). The reverse Warburg effect: aerobic glycolysis in cancer associated fibroblasts and the tumor stroma. Cell Cycle.

[18] Fiori ME (2019). Cancer-associated fibroblasts as abettors of tumor progression at the crossroads of EMT and therapy resistance. Mol. Cancer.

[19] Fei F, Qu J, Zhang M, Li Y, Zhang S (2017). S100A4 in cancer progression and metastasis: a systematic review. Oncotarget.

[20] Sasaki K (2018). Analysis of cancer-associated fibroblasts and the epithelial-mesenchymal transition in cutaneous basal cell carcinoma, squamous cell carcinoma, and malignant melanoma. Hum. Pathol..

[21] Li F (2018). S100A4-MYH9 axis promote migration and invasion of gastric cancer cells by inducing TGF-β-mediated epithelial-mesenchymal transition. J. Cancer.

[22] Zhai X (2014). Abnormal expression of EMT-related proteins, S100A4, vimentin and E-cadherin, is correlated with clinicopathological features and prognosis in HCC. Med. Oncol..

[23] Liu M (2017). Inversed expression patterns of S100A4 and E-cadherin in cervical cancers: implication in epithelial-mesenchymal transition. Anat. Rec. (Hoboken).

[24] Shangguan C (2018). Cancer-associated fibroblasts enhance tumor (18)F-FDG uptake and contribute to the intratumor heterogeneity of PET-CT. Theranostics.

[25] Schmidt KC, Smith CB (2005). Resolution, sensitivity and precision with autoradiography and small animal positron emission tomography: implications for functional brain imaging in animal research. Nucl. Med. Biol..

[26] Huang T (2012). Tumor microenvironment-dependent 18F-FDG, 18F-fluorothymidine, and 18F-misonidazole uptake: a pilot study in mouse models of human non-small cell lung cancer. J. Nucl. Med..

[27] Caro LG, Van Tubergen RP, Kolb JA (1962). High-resolution autoradiography. I. Methods. J. Cell Biol..

[28] Yamato M, Kataoka Y, Mizuma H, Wada Y, Watanabe Y (2009). PET and macro- and microautoradiographic studies combined with immunohistochemistry for monitoring rat intestinal ulceration and healing processes. J. Nucl. Med..

[29] Aguilar-Cazares D (2019). Contribution of angiogenesis to inflammation and cancer. Front. Oncol..

[30] Lyssiotis CA, Kimmelman AC (2017). Metabolic interactions in the tumor microenvironment. Trends Cell Biol..

[31] Wu SC (2016). Bispecific antibody conjugated manganese-based magnetic engineered iron oxide for imaging of HER2/neu- and EGFR-expressing tumors. Theranostics.

[32] Liu B (2019). Hypoxia-induced autophagy promotes EGFR loss in specific cell contexts, which leads to cell death and enhanced radiosensitivity. Int. J. Biochem. Cell Biol..

[33] Abdel-Wahab AF, Mahmoud W, Al-Harizy RM (2019). Targeting glucose metabolism to suppress cancer progression: prospective of anti-glycolytic cancer therapy. Pharmacol. Res..

[34] Warburg O, Wind F, Negelein E (1927). The metabolism of tumors in the body. J. Gen. Physiol..

[35] Amoedo ND, Obre E, Rossignol R (2017). Drug discovery strategies in the field of tumor energy metabolism: limitations by metabolic flexibility and metabolic resistance to chemotherapy. Biochim. Biophys. Acta Bioenerg..

[36] Petrova V, Annicchiarico-Petruzzelli M, Melino G, Amelio I (2018). The hypoxic tumour microenvironment. Oncogenesis.

[37] Shen B, Huang T, Sun Y, Jin Z, Li XF (2017). Revisit 18F-fluorodeoxyglucose oncology positron emission tomography: "systems molecular imaging" of glucose metabolism. Oncotarget.

[38] Higashi K (2000). Correlation of Glut-1 glucose transporter expression with [18F]FDG uptake in non-small cell lung cancer. Eur. J. Nucl. Med..

[39] Masoud GN, Li W (2015). HIF-1α pathway: role, regulation and intervention for cancer therapy. Acta Pharm. Sin. B.

[40] Lee CM, Tannock IF (2010). The distribution of the therapeutic monoclonal antibodies cetuximab and trastuzumab within solid tumors. BMC Cancer.

[41] Milik SN (2018). Surmounting the resistance against EGFR inhibitors through the development of thieno[2,3-d]pyrimidine-based dual EGFR/HER2 inhibitors. Eur. J. Med. Chem..

[42] Takezawa K (2012). HER2 amplification: a potential mechanism of acquired resistance to EGFR inhibition in EGFR-mutant lung cancers that lack the second-site EGFRT790M mutation. Cancer Discov..

[43] Yoshida T (2016). Standardized uptake value on (18)F-FDG-PET/CT is a predictor of EGFR T790M mutation status in patients with acquired resistance to EGFR-TKIs. Lung Cancer.

[44] Baker LCJ (2018). Evaluating imaging biomarkers of acquired resistance to targeted EGFR therapy in Xenograft models of human head and neck squamous cell carcinoma. Front. Oncol..

[45] Rajendran JG (2004). Hypoxia and glucose metabolism in malignant tumors: evaluation by [18F]fluoromisonidazole and [18F]fluorodeoxyglucose positron emission tomography imaging. Clin. Cancer Res..

[46] Crispin-Ortuzar M (2018). Predicting hypoxia status using a combination of contrast-enhanced computed tomography and [(18)F]-Fluorodeoxyglucose positron emission tomography radiomics features. Radiother. Oncol..

[47] Misra A, Pandey C, Sze SK, Thanabalu T (2012). Hypoxia activated EGFR signaling induces epithelial to mesenchymal transition (EMT). PLoS ONE.

[48] Fu Y (2017). The reverse Warburg effect is likely to be an Achilles' heel of cancer that can be exploited for cancer therapy. Oncotarget.

[49] Primeau AJ, Rendon A, Hedley D, Lilge L, Tannock IF (2005). The distribution of the anticancer drug Doxorubicin in relation to blood vessels in solid tumors. Clin. Cancer Res..

[50] Goel S (2011). Normalization of the vasculature for treatment of cancer and other diseases. Physiol. Rev..

[51] Hamacher K, Coenen HH, Stöcklin G (1986). Efficient stereospecific synthesis of no-carrier-added 2-[18F]-fluoro-2-deoxy-D-glucose using aminopolyether supported nucleophilic substitution. J. Nucl. Med..

