# Supplementary material for: Involvement of cancer-derived EMT cells in the accumulation of 18F-fluorodeoxyglucose in the hypoxic cancer microenvironment
Source: Sci Rep. 2021 May 17;11:9668. doi: 10.1038/s41598-021-88414-1 (PMC8126561; doi:10.1038/s41598-021-88414-1)
Supplement: Supplementary file 1 — Supplementary Information [file 41598_2021_88414_MOESM1_ESM.pdf]

Involvement of cancer-derived EMT cells in the accumulation of ^{18}F-fluorodeoxyglucose in the hypoxic cancer microenvironment

Sachi Sugita[1,2], Masanori Yamato[2,3], Toshimitsu Hatabu[1] and Yosky Kataoka[2,3]*

[1] Laboratory of Animal Physiology, Graduate School of Environmental and Life Science, Okayama University, Okayama, Okayama 700-8530, Japan.

[2] Laboratory for Cellular Function Imaging, RIKEN Center for Biosystems Dynamics Research, 6-7-3 Minatojima-minamimachi, Chuo-ku, Kobe, Hyogo 650-0047, Japan.

[3] Multi-modal Microstructure Analysis Unit, RIKEN-JEOL Collaboration Center, 6-7-3 Minatojima-minamimachi, Chuo-ku, Kobe, Hyogo 650-0047, Japan.

*Correspondence should be addressed to: Yosky Kataoka

Laboratory for Cellular Function Imaging, RIKEN Center for Biosystems Dynamics Research, 6-7-3 Minatojima-minamimachi, Chuo-ku, Kobe, Hyogo 650-0047, Japan

E-mail: kataokay@riken.jp

TEL: +81-78-304-7115

FAX: +81-78-304-7161

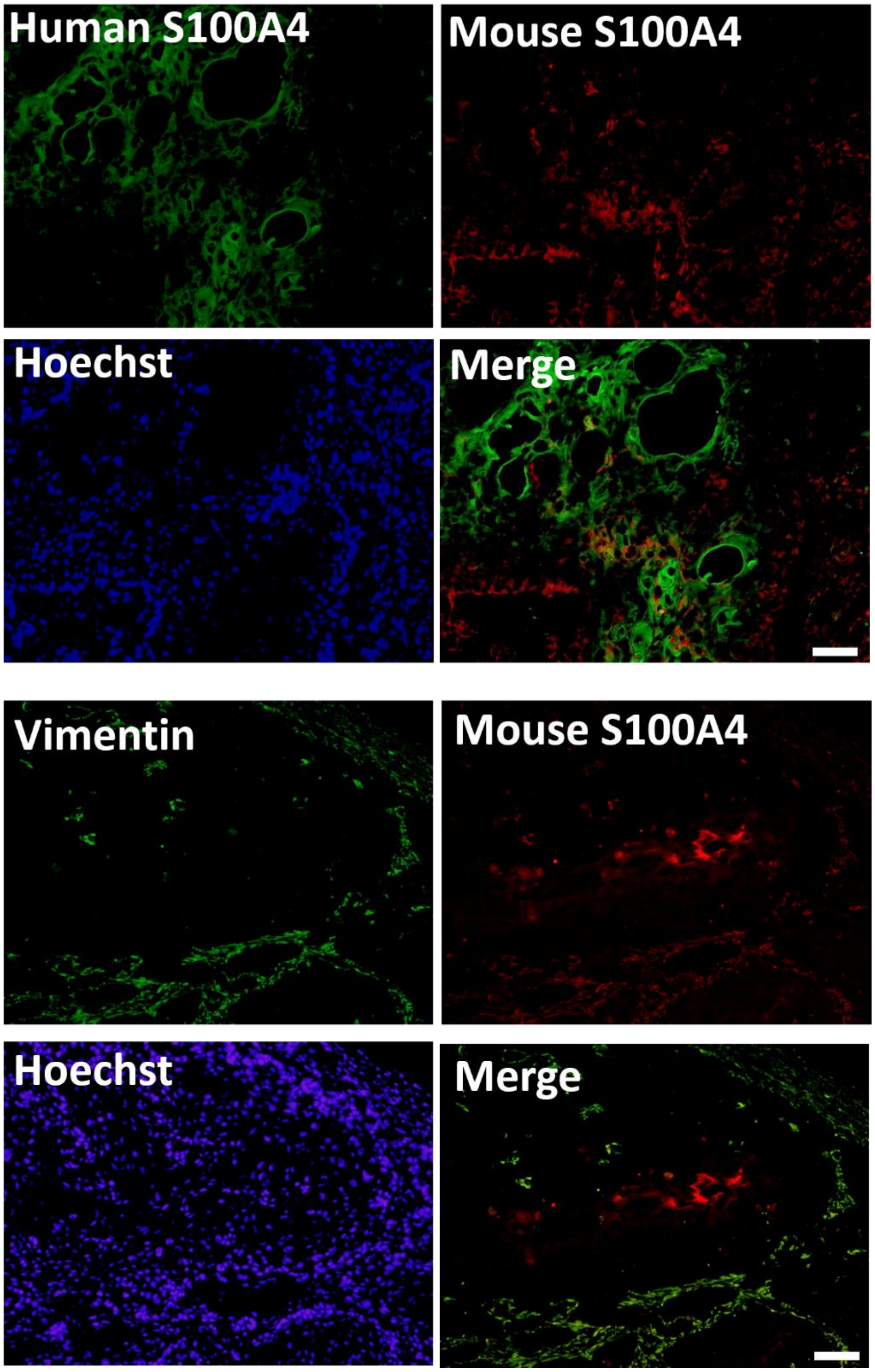

Supplementary Fig. 1 Human S100A4, Mouse S100A4, **and vimentin expression patterns in A431 tumor cells**. Bars: 100 µm

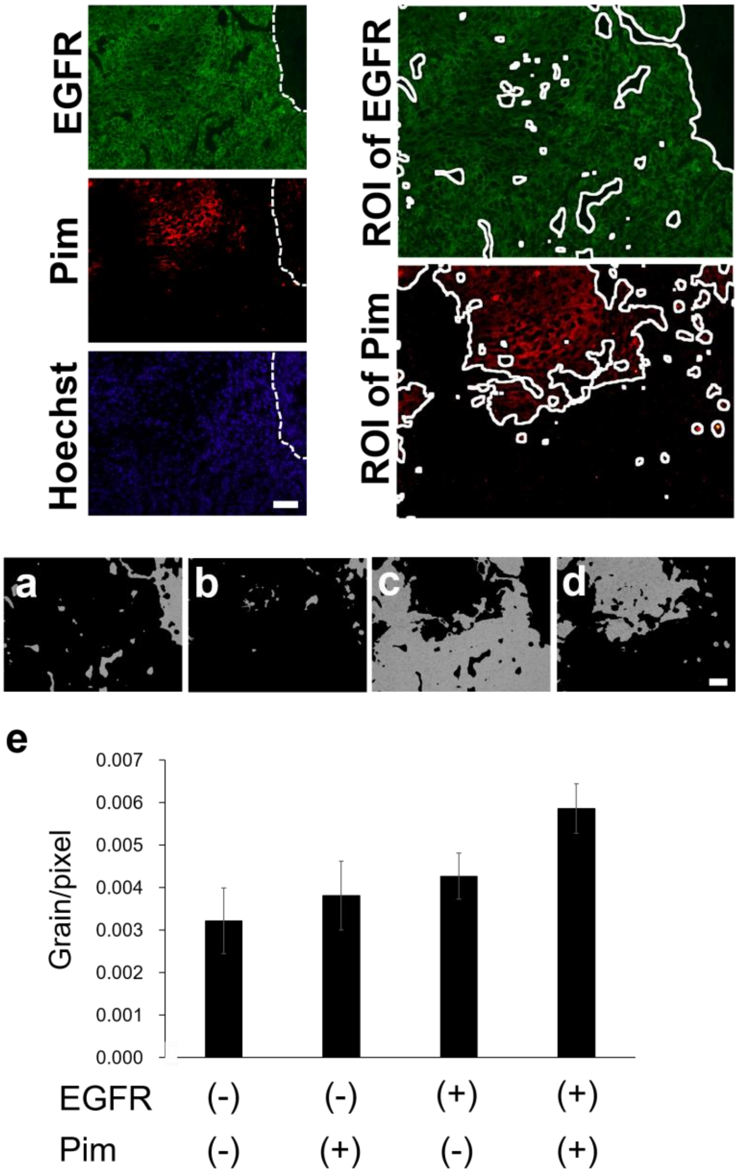

Supplementary Fig. 2 ^{18}F-FDG accumulation in hypoxic cells expressing EGFR. The dotted line shows the boundary between the tumor and the skin area. The white line shows the computer determined ROI. a) ROI image showing the pimonidazole- and EGFR-negative area, b) pimonidazole-positive and EGFR-negative area, c) pimonidazole-negative and EGFR-positive area, and d) pimonidazole- and EGFR-positive area. e) Determination of the grain density in the pimonidazole- and EGFR-positive or negative area in each ROI (n = 3, mean ± SEM). Pim: Pimonidazole. Bars: 100 µm

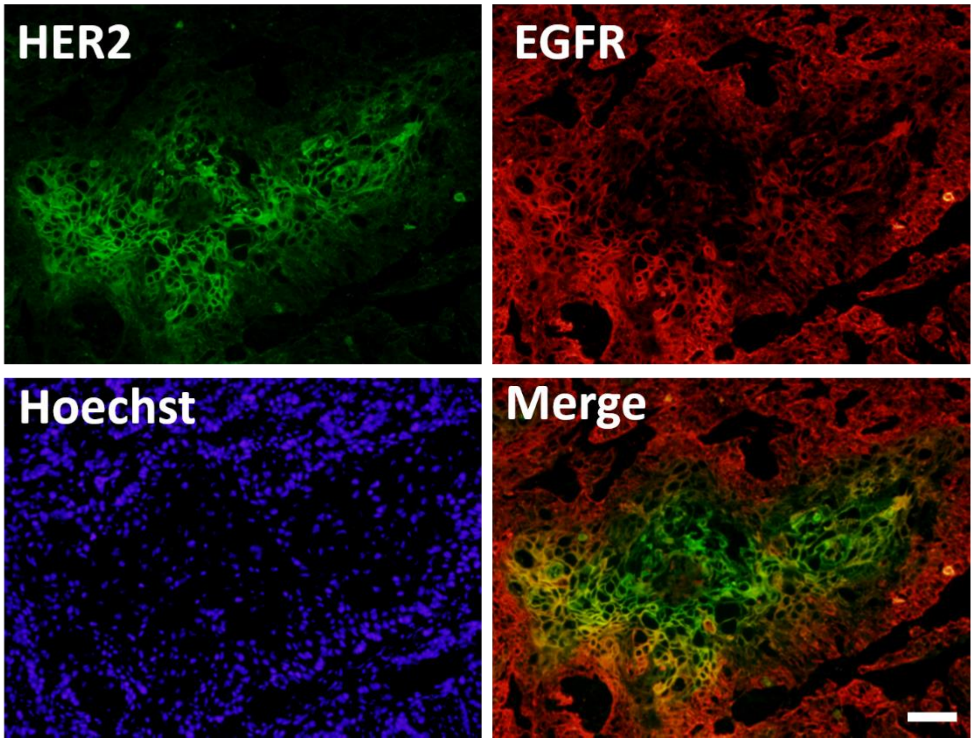

Supplementary Fig. 3 EGFR and HER2 expression patterns in A431 tumor cells. Bar: 100 μm

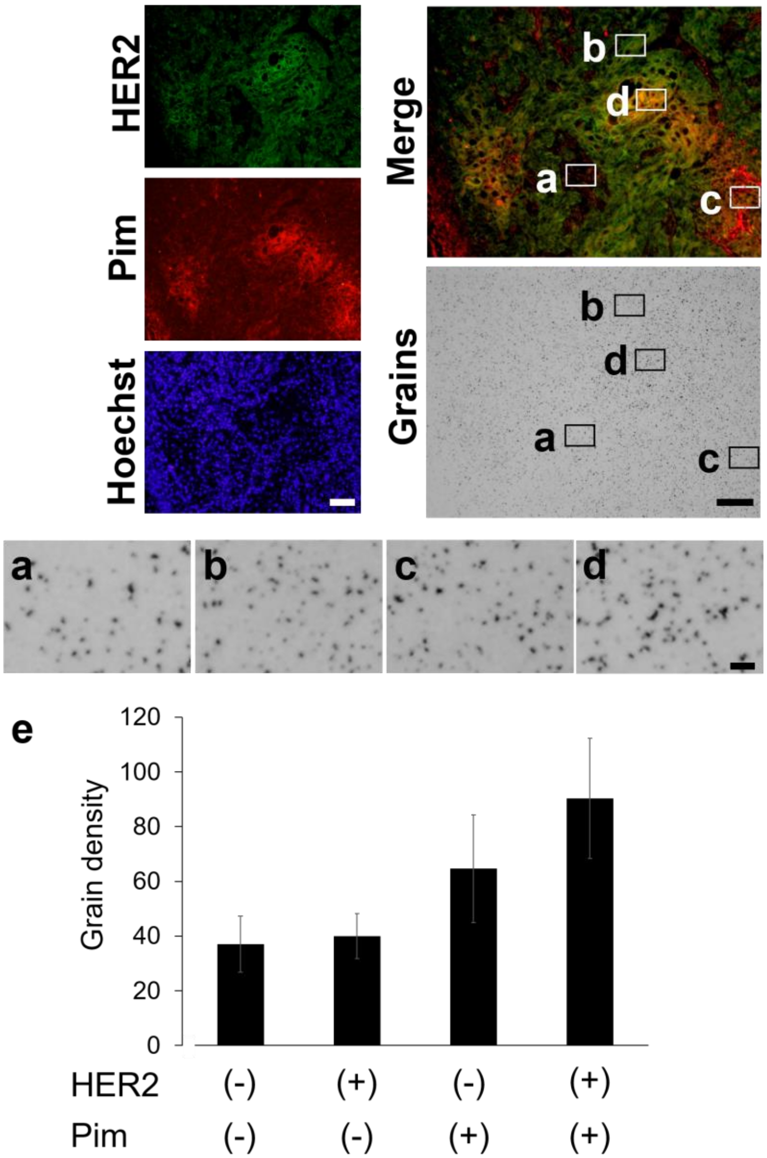

Supplementary Fig. 4 ^{18}F-FDG accumulation in hypoxic cells expressing HER2. a) Magnified view of the micro-autoradiographic image showing the pimonidazole- and HER2-negative area, b) pimonidazole-negative and HER2-positive area, c) pimonidazole-positive and HER2-negative area, and d) pimonidazole- and HER2-positive area. e) Determining the grain density in the pimonidazole- and HER2-positive and negative areas by focusing on ROIs of the same size (n = 3, mean ± SEM). Pim: Pimonidazole. Bars: 100 μm (HER2, Pimonidazole, Hoechst, Merge and Grains), 10 μm (a-d)

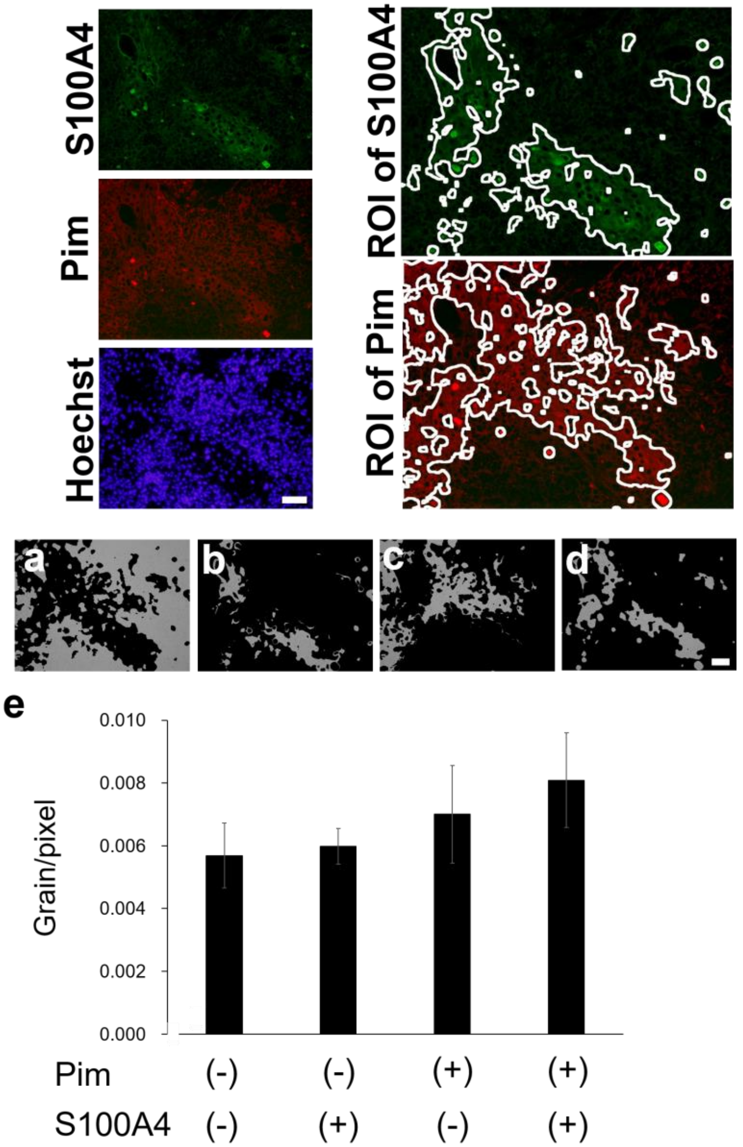

Supplementary Fig. 5 ^{18}F-FDG accumulation in hypoxic tumor cells expressing human S100A4. The white line shows the computer-determined ROI. a) Merged micro-autoradiography and ROI image showing the pimonidazole- and human S100A4-negative area, b) pimonidazole-negative and human S100A4-positive area, c) pimonidazole-positive and human S100A4-negative area, and d) pimonidazole- and human S100A4-positive area. e) Grain density in the pimonidazole- and human S100A4-positive or negative area in each ROI (n = 3, mean ± SEM). Pim: Pimonidazole. Bars: 100 µm

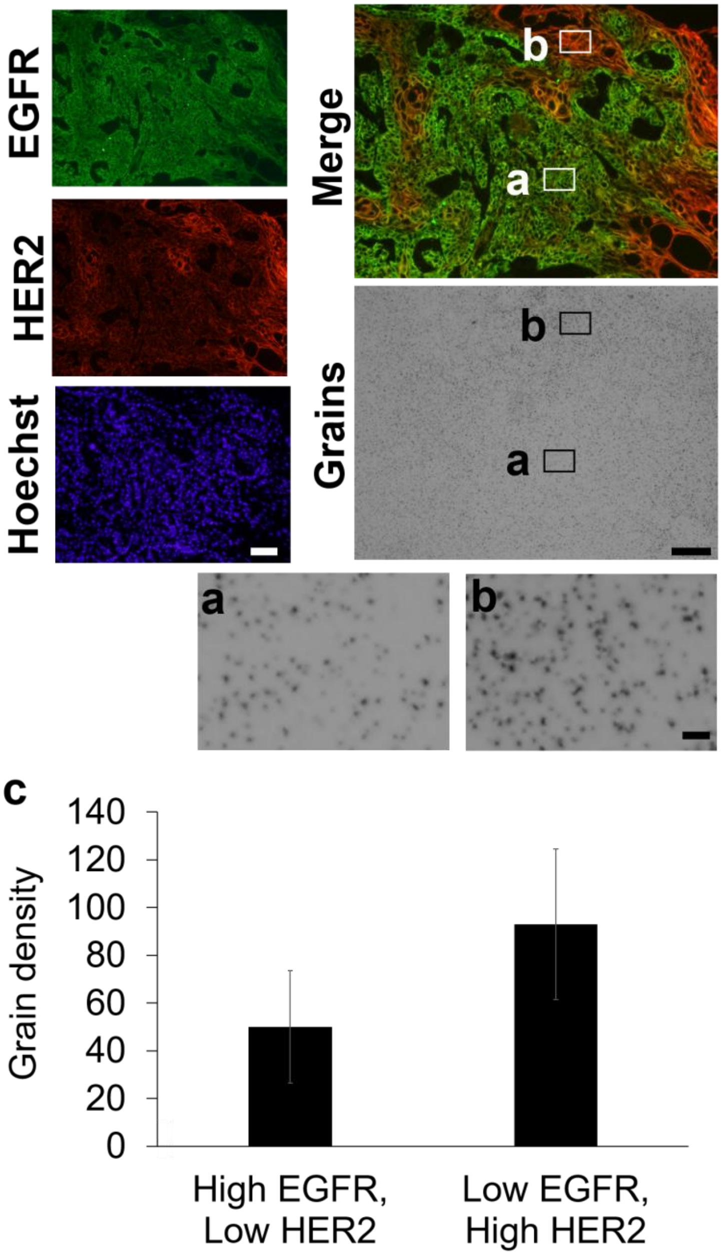

Supplementary Fig. 6 ^{18}F-FDG accumulation in tumor cells expressing EGFR or HER2.

The ROI images (a, and b) show the regions with intensively stained EGFR and weakly stained HER2 (high EGFR/low HER2, a) and with weakly stained EGFR and intensively stained HER2 (low EGFR/high HER2, b). c) Grain density in the region with high EGFR/low HER2 or low EGFR/high HER2 (n = 3, mean ± SEM). Pim: Pimonidazole. Bars: 100 μm. (EGFR, HER2, Hoechst, Merge and Grains), 10 μm (a, b)

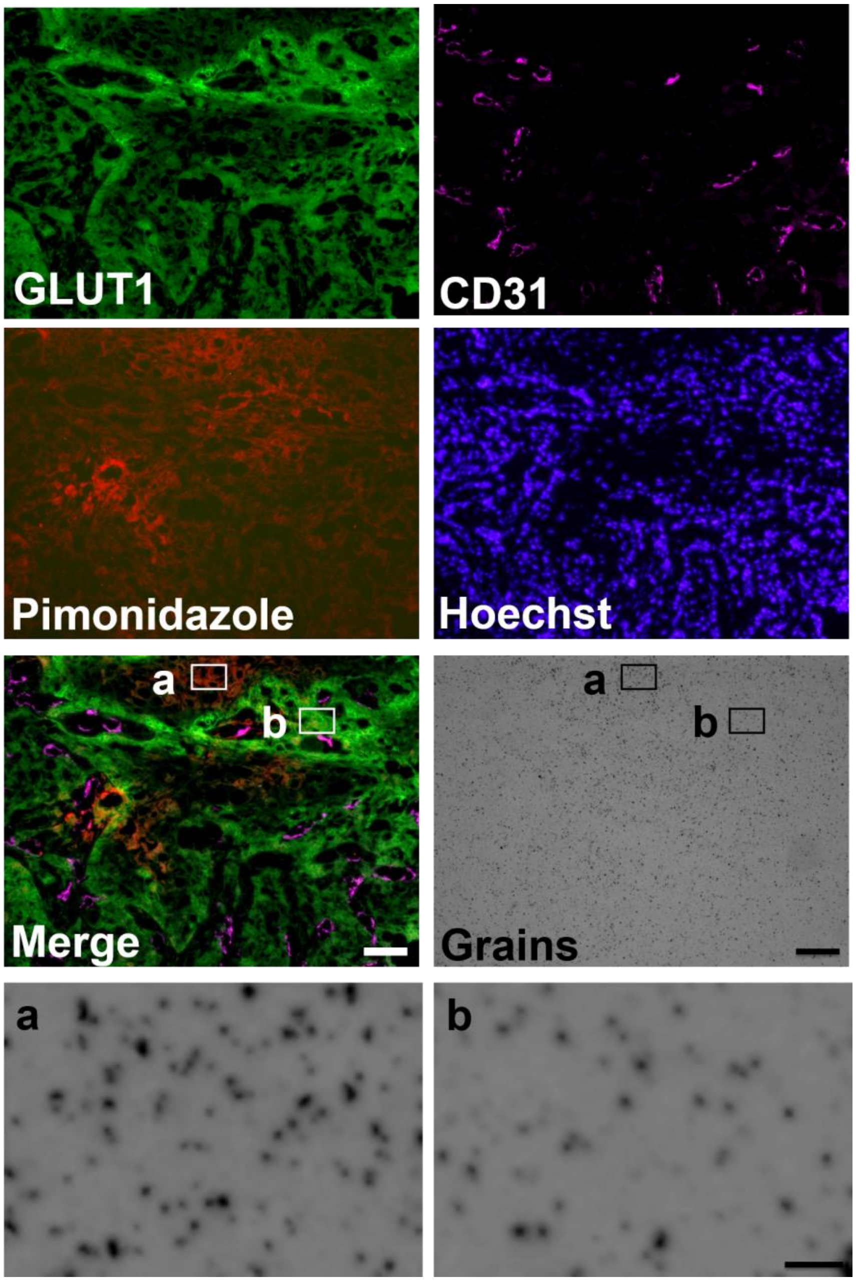

Supplementary Fig. 7 ^{18}F-FDG accumulation in tumor cells expressing GLUT1 and Pimonidazole. The ROI images (a, and b) show the regions with intensively stained pimonidazole and weakly stained GLUT1 (a) and with weakly stained pimonidazole and intensively stained GLUT1 (b). Bars: 100 μm (GLUT1, Pimonidazole, CD31, Hoechst, Merge and Grains), 10 μm (a, b)
